# Sugarcane Omics: An Update on the Current Status of Research and Crop Improvement

**DOI:** 10.3390/plants8090344

**Published:** 2019-09-12

**Authors:** Ahmad Ali, Mehran Khan, Rahat Sharif, Muhammad Mujtaba, San-Ji Gao

**Affiliations:** 1 National Engineering Research Center for Sugarcane, Fujian Agriculture and Forestry University, Fuzhou 350002, China; 2 Department of Plant Protection, Faculty of Agricultural Sciences, Ghazi University, Dera Ghazi Khan, Punjab 32200, Pakistan; 3 College of Horticulture, Northwest A&F University, Yangling 712100, China; 4 Institute of Biotechnology, Ankara University, Ankara 06110, Turkey

**Keywords:** sugarcane, omics approaches, biotic and abiotic stresses, crop improvement and development

## Abstract

Sugarcane is an important crop from Poaceae family, contributing about 80% of the total world’s sucrose with an annual value of around US$150 billion. In addition, sugarcane is utilized as a raw material for the production of bioethanol, which is an alternate source of renewable energy. Moving towards sugarcane omics, a remarkable success has been achieved in gene transfer from a wide variety of plant and non-plant sources to sugarcane, with the accessibility of efficient transformation systems, selectable marker genes, and genetic engineering gears. Genetic engineering techniques make possible to clone and characterize useful genes and also to improve commercially important traits in elite sugarcane clones that subsequently lead to the development of an ideal cultivar. Sugarcane is a complex polyploidy crop, and hence no single technique has been found to be the best for the confirmation of polygenic and phenotypic characteristics. To better understand the application of basic omics in sugarcane regarding agronomic characters and industrial quality traits as well as responses to diverse biotic and abiotic stresses, it is important to explore the physiology, genome structure, functional integrity, and collinearity of sugarcane with other more or less similar crops/plants. Genetic improvements in this crop are hampered by its complex genome, low fertility ratio, longer production cycle, and susceptibility to several biotic and abiotic stresses. Biotechnology interventions are expected to pave the way for addressing these obstacles and improving sugarcane crop. Thus, this review article highlights up to date information with respect to how advanced data of omics (genomics, transcriptomic, proteomics and metabolomics) can be employed to improve sugarcane crops.

## 1. Introduction

Sugarcane is the main supplier of world sugar, accounting for 80% of global sugar production (http://www.sucden.com/). In addition, it has the excessive potential for bioethanol production, and about 50% of cane is used to produce ethanol in Brazil [1,2]. Modern sugarcane varieties are a complex genome, high polyploidy and aneuploidy resulted from about 70%–80% of the genome composition from *Saccharum officinarum*, and 10%–20% comes from *S. spontaneum* [3], combining the high sugar content of *S. officinarum* with the hardiness, disease resistance, and ratooning of *S. spontaneum* [4,5]. Sugarcane improvement based on conventional breeding is highly challenging due to this crop posing narrow genetic pools and a complicated genome [6]. Recently, numerous researches about molecular biology have been investigated in sugarcane, including cytogenetic analysis and omics research (genomics, transcriptomics, proteomics, and metabolomics) in order to achieve higher yields, higher sucrose content, and biotic and abiotic stress tolerance, as well as to understand their genetic regulation and mechanisms [6,7,8,9].

The omics approaches benefit from the understanding of the complex connections among genetic makeup, genes, proteins, and metabolites, but they rely seriously on analytical methods, such as bioinformatics, computational analysis, etc., and many other disciplines of biology [6]. A great amount of new information has been obtained regarding the molecular mechanisms of sugarcane resistance and tolerance to herbicides, cold, drought, and salinity stress, as well as plant development [7,8]. In early genomic research, molecular marker approaches helped to elucidate the genome structure of modern sugarcane genotypes and derive phylogenetic relationships among the *Saccharum* complex; Also, much work was carried out on sugarcane genome mapping experiments to detect marker-trait associations and to validate the position of different essential genes [6,10]. In recent decades numerous transcriptomic experiments have led to the identification of a large number of genes which are involved in controlling critical biological functions [6,11]. The genes identified through transcriptomic approaches could be used either as DNA markers or to develop transgenic sugarcane [6,12]. In addition, various differentially expressed proteins (DEPs) and their roles in signal transduction pathway response to biotic and abiotic stresses were revealed by proteomic approaches, such as two-dimensional difference gel electrophoresis (2D-DIGE) [7] and isobaric tags for relative and absolute quantitation (iTRAQ) [13]. More recently, metabolite analysis provides a deeper understanding of the complex regulatory processes of potential metabolites (such as saccharides and other derivatives) and predicts resistance mechanisms through the use of high-throughput technologies that can determine metabolic phenotypes [14,15].

**Figure 1:**
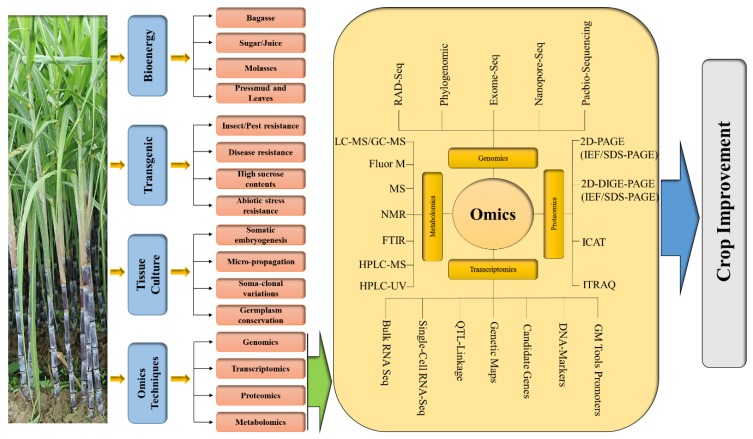
Graphical strategy showing the role of biotechnological interventions for the development of sugarcane crop. Abbreviations: LC, Liquid chromatography; MS, Mass spectrometry; GC, Gas chromatography; Flour-M, Gas chromatography; NMR, Nuclear magnetic resonance; FTIR, Fourier-transform infrared spectroscopy; HPLC, High-performance liquid chromatography; UV, Ultraviolet light; SDS, Sodium dodecyl sulfate; PAGE, polyacrylamide gel; 2D-DIGE, Two-dimensional difference gel electrophoresis; iTRAQ, Isobaric tags for relative and absolute quantitation; QTL, Quantitative trait loci; GM, Genetics modification; ICAT, Isotope-coded affinity tag; RAD-Seq, Restriction site-associated DNA sequencing.

Omics is a genuinely revolutionary area of upcoming research that has been outlined to spotlight the usage of genomics, transcriptomics, proteomics, and metabolomics in sugarcane crop improvement and energy cane development (Figure 1). Thus, this review provides a complete overview of the latest directions and developments in omics technologies and their uses in this crop improvement.

## 2. Sugarcane Genomics

The modern sugarcane cultivar genome has not been assembled until now due to its complexity of the interspecific, polyploid, and aneuploid nature [5]. The monoploid genome size of *S. officinarum* is 930 Mb (mega base pairs) and that of *S. spontaneum* is 750 Mb, twofold the size of the *Oryza sativa* genome (~390 Mb) [6]. More recently, Zhang et al. [4] assembled the autopolyploid sugarcane *S. spontaneum* genome, using haploid *S. spontaneum* (AP85-441), enabling the association of 32 pseudo-chromosomes holding eight homologous groups of four members each, containing 35,525 genes with alleles defined. The reduction in the number of essential chromosomes from *S. spontaneum* from ten to eight was caused by the fission of two ancestral chromosomes and the subsequent translocation to four chromosomes. Remarkably, 80% of the nucleotide-binding site encoding genes related with disease resistance were spotted in four rearranged chromosomes and 51% were positioned in rearranged regions. Additionally, Garsmeur et al. [5] generated a bacterial artificial chromosome (BAC)-based sugarcane monoploid genome sequence with the sorghum genome as a reference. Based on genome-wide analysis, the minimal tiling path of the 4,660 cane BAC that best covered the sorghum genomic gene-rich portion was selected, sequenced, and assembled in a 382-Mb single tiling pathway of a high quality sequence with the total production of 25,316 protein-coding gene models, of which 17% showed no collinearity with their sorghum orthologs. Their findings show that the two species *S. officinarum* and *S. spontaneum* involved in modern cultivars differ in their genomic size and different basic chromosome numbers due to their transposable elements (TEs) and some large chromosomal rearrangements. Geneticists are trying to explain the link between genomes of the complex sugarcane and other comparatively similar crop/plants. The genome level of plants in Poaceae varies from diploid to triploid [16]. The preservation and origin of gene function are caused by the gene sequence, and the gene sequence is sustained by the homology of the genome [17]. The genome expansion in grasses strongly supports the TE mainly between coding genes [18,19]. Transposons and retrotransposons are the two types of TE. Moreover, in plants, the most copious reverse transcription element is the LTR (long terminal repeat) retrotransposon [6,20]. Transposase proteins are involved in the insertion–deletion mechanism. The active site of transcription controls the movement of the retrotransposon, which is reinserted into the genome after each breeding cycle to increase the copy number [20].

Recent studies have shown that there are mechanisms for gene remodeling that lead to the production of new genes. Due to genetic remodeling, new regulatory networks have altered gene expression [21]. Studies on TEs in wheat and barley [22,23] provide a close relationship between TEs and genomic structures. In sugarcane, TEs can be activated and assessed by functional transcriptomic approaches [24]. Therefore, grasses with a broad genome, especially sugarcane, have a certain degree of TEs, which can be triggered and analyzed step by step by functional transcription techniques [25]. This can reveal the complexity of sugarcane traits for example accumulation of sucrose, fiber content and pathogen-resistance proteins research. Recent studies have shown that mutant-like transposases are the most descriptive transposon transcripts in the sugarcane transcriptome [17]. Sugarcane reverse genetics studies and transcriptome analysis, confirmed that mutant-like transposons provide at least four (I–IV) groups of evidence in monocots/true dicots [26].

Despite the genomic complexity of sugarcane, genome-wide association studies (GWAS) may be used to identify marker-trait associations (MTAs) and then assist breeders in better managing crosses and selecting superior genotypes in breeding programs. For example, Yang et al. [27] identified resistance loci to orange rust and yellow leaf virus diseases in sugarcane through GWAS [27] and Barreto et al. [28] reported 23 MTAs related to soluble solid content, stalk height, stalk number, stalk weight, and cane yield traits in sugarcane (*Saccharum* spp.) using GWAS with a multi-locus mixed model [28]. In addition, an allele-defined genome of *S. spontaneum* is available [4], offering the opportunity to perform the identification and phylogeny of various gene families in sugarcane at a genome-wide scale. Moreover, the expression patterns of these gene families can also be determined by RNA-Seq data [29].

## 3. Sugarcane Transcriptomics

Transcriptome analysis provides the required data regarding genes through various in silico techniques, including probe hybridization array, expressed sequenced tags (ESTs), or known genes of other allied crops. Brazilian sugarcane EST database is known as one of the largest as it contains around 238,000 ESTs collected from 26 different cDNA libraries that were constructed using different tissue from a large set of Brazilian varieties [30,31,32]. The ESTs were organized intro 43,141 putative unique transcripts having 26,803 contigs and 16,338 singletons, all collectively stated as sugarcane assembled sequences [30]. The sugarcane gene index (version 3.0) comprises of 282,683 ESTs and a set of 499 cDNA sequences with 121,342 unigenes. However, there are around 10,000 unidentified sugarcane coding genes [33].

Recently, the sequencing results of transcriptome from 59 F1 individuals (*S. officinarum* LA Purple and *S. robustum*) resulted in 11,157 and 8,998 single nucleotide polymorphisms (SNPs) and 83 and 105 linkage groups, respectively [34]. However, the lack of a reference accurate and integral sugarcane genome is a challenge for gene function prediction and utilization of the transcriptome dataset [33]. Thus, the reference genome of *Sorghum bicolor* is commonly used in sugarcane transcriptome studies due to the higher homology (95%) in the genic regions between sugarcane and sorghum genomes [35,36]. Among the BLASTx top hits, ~47% unigenes of sugarcane transcriptome data were matched to *Sorghum bicolor* proteins, but only ~2% unigenes showed significant homology with those of the sugarcane hybrid cultivar R570, suggesting the high genetic variation among different sugarcane genotypes [33]. 

**Figure 2:**
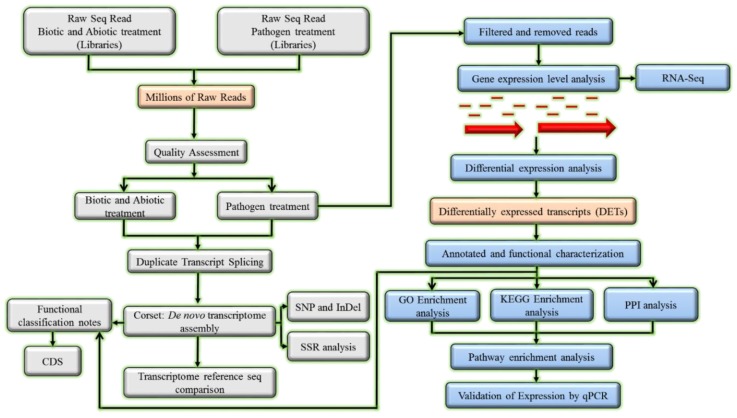
A workflow sketch of sugarcane transcriptome analysis including construction of reference transcriptome from the de novo assembly and annotation and functional characterization of differentially expressed transcripts (DETs).

High-throughput RNA-Seq has been widely used in eukaryotic transcriptome analyses [37]. However, short reads arising from second-generation sequencing technologies require very large computational assemblies and cannot span full-length transcripts, resulting in the accuracy reduction of gene model prediction [38]. Thus, single-molecule long-read sequencing technology, such as Pacific Biosciences long-read isoform sequencing (Iso-Seq), has been developed and widely used in transcriptome sequencing because this technique offers a better alternative for sequencing more complete transcriptomes and successfully predicting and validating gene models [38]. The Iso-Seq approach also has been applied to the long-read transcriptome of sugarcane [39,40]. Figure 2 shows the workflow sketch of sugarcane transcriptome analysis. 

### 3.1. Transcriptomics Studies on Sugarcane Response to Biotic Stresses

Plants developed complex protective approaches against various biotic stresses including diseases and insects. Between sugarcane and fungi interaction, Muthiah et al. [41] investigated the possible role of transcription factors (TFs) in the regulation of defense responses against *Colletotrichum falcatum* causing red rot in sugarcane. Five different groups of TFs (bZIP, MYB, WRKY, NAC, and TLP) were screened by conducting two parallel experiments for differential expression. Among the studies of 41 TFs, differential regulation of 24 TFs after pathogen challenge and differential regulation of 15 TFs after systemic acquired resistance (SAR) inducer induction were perceived. Overall, the results suggest that early induction of TF may involve actively prompting or coordinating resistance against pathogen attack. Sathyabhama et al. [42] investigated the ESTs generated after inoculating two sugarcane cultivars resistant (cv. Co 93009) and susceptible (cv. CoC 671) using the inoculum of *C. falcatum*. The differential expression was estimated by carrying out the enriched forward subtraction. At the last step of subtraction, 136 EST sequences were assembled into 10 clusters through cloning and sequencing. It was found that these clusters are involved in plant reactive oxygen species signaling, defense and secretion pathways, and are involved in allergic reaction-mediated programmed cell death. Prasanth et al. [43] generated a large set of transcript reads (24,732) specific to *C. falcatum* predicting around 13,320 genes. The virulence genes have been categorized into candidate effectors, transition specific and transporters, secondary metabolites, proteases and peptidases which showed that the transcript of *C. falcatum* encodes a huge number of secondary metabolites and membrane transporters. More recently, comparative transcriptome analysis of candidate secretory effector proteins from *C. falcatum* infecting sugarcane revealed that these predicted secretory proteins have a probable role in stabilizing fungal secretory proteins in the host system during pathogenesis [44].

Smut caused by *Sporisorium scitamineum* is one of the important diseases affecting sugarcane. Regarding the interaction between sugarcane and this pathogen, Huang et al. [45], based on the differential expression data achieved from suppression subtractive hybridization (SSH) libraries and qRT-PCR, revealed some major pathways as response components to *S. scitamineum* stress in sugarcane, such as serine/threonine kinases, Ca^2+^ sensors, mitogen-activated protein genes, some Nucleotide-binding site leucine-rich repeat (NBS-LRR) genes, and particularly in the genes (auxin, abscisic acid, salicylic acid and ethylene) related to plant hormone signaling pathways. McNeil et al. [46] also revealed that some differentially expressed genes (DEGs) involved in the phenylpropanoid pathway, cell wall biosynthesis, plant hormone signal transduction and disease resistance genes.

Brown rust caused by *Puccinia melanocephala* is another important disease of sugarcane. Putative resistance-associated genes of 11 out of 217 unigenes from the subtractive library were induced in sugarcane in response to this pathogen [47]. In addition, *Fusarium verticillioides* is associated with pokkah boeng disease. Lin et al. [48] revealed that a total of 1,779 transcripts out of 13,999 annotated genes were differentially expressed in *F. verticillioides* (CNO-1) grown in the different sources of nitrogen. These transcripts were involved in nitrogen metabolism, transport, and assimilation, and several transcription factors were related to nitrogen utilization in biological processes while numerous genes were associated with pathogenicity. More recently, Wang et al. [49] identified that major DEGs involved in resistance were significantly related to metabolic pathways of phenylpropanoid biosynthesis, cutin, suberine and wax biosynthesis, nitrogenous metabolism, and plant–pathogen interactions under two cultivars “YT 94/128” (resistant) and “GT 37” (susceptible) inoculated with *F. verticillioides*. 

Between the sugarcane-bacteria interaction, Santa Brigida et al. [50] identified that 467 DEPs and several metabolic pathways in sugarcane in response to infection by *Acidovorax avenae* subsp. *avenae*, a causal pathogen of red stripe. Further, differential analysis revealed that some genes were upregulated, such as genes in the biosynthetic pathways of Ethylene (ET) and Jasmonate (JA) pattern recognition receptors (PRRs), oxidative burst genes, NBS-LRR genes, cell wall fortification genes, SAR induced genes and pathogenesis-related genes (PR). Another important bacterial disease of sugarcane is the ratoon stunting disease caused by bacterial pathogen of *Leifsonia xyli* subsp. *xyli* (*Lxx*). Zhang et al. [51] revealed that sugarcane infection with *Lxx* induced changes in the production of auxin (IAA), gibberellic acid (GA3), and abscisic acid (ABA). Zhu et al. [52] found that plant height, stalk diameter, single stalk weight, and water potential of *Lxx*-infected sugarcane plants were decreased while membrane permeability and amino acid content were increased compared to the control. In addition, the expression of phenylalanine ammonia-lyase (PAL), zinc finger protein (ZFP) and nucleotide-binding site leucine-rich repeat (NBS-LRR) genes all also increased in plants response to *Lxx* infection. Subsequently, Zhu et al. [53] performed functional analysis of a membrane protein gene *Lxx18460* (anti-sigma K) that was transformed into *Nicotiana tabacum*, and proposed that *Lxx 18460* has an adverse impact on the growth of tobacco, reducing the photosynthesis of tobacco, destroying the activity of defense enzymes, and affecting the levels of endogenous hormones. Cia et al. [54] identified 267 DEGs and 150 proteins involved in plant growth, hormone metabolism, signal transduction, and defense responses that were affected after sugarcane infection by *Lxx* pathogen. 

A few studies depict about sugarcane-viruses interaction based on the RNA-seq data. *Sorghum mosaic virus* and *Sugarcane steak mosaic virus* (SCSMV) are two main pathogens causing sugarcane mosaic disease in China [55]. Recently, Dong et al. [56] demonstrated that 3,791 DEGs were upregulated and 50 DEGs were downregulated, and the three main KEGG pathways, ubiquitin proteolytic system, proteasome and translational pathways in endoplasmic reticulum in sugarcane cultivars under SCSMV infection. Additionally, Lin et al. [57] found that 481 DEGs and 51 homologous sequences of potyvirus host interactor (PHI) genes from RNA-seq data, implying that the endoplasmic reticulum, some defense related genes, and Ca^2+^, reactive oxygen species (ROS), cytokinin, auxin, and ethylene signaling, a calmodulin-related protein gene, and an ethylene-inducible TF gene were associated with regulations in sugarcane response to SrMV infection. These studies may help to understand the molecular mechanisms underlying sugarcane-virus interaction.

### 3.2. Transcriptomics Studies on Sugarcane Response to Abiotic Stresses

The investigation of transcriptomic responses of sugarcane against the abiotic factors can give an insight into the defense mechanisms and possible molecular strategies for developing resistant varieties. The role of TFs (WRKY, MYB, bZIP, AP2/DREBP, and zinc finger-like proteins) is well studied in plants acting as regulatory agents of metabolic pathways responsible for plant defenses against environmental stresses [58]. Abiotic stress factors (drought, cold and high salinity, etc.) adversely affect plant growth and hence productivity and drought stress has been considered as one of the most limiting factors in sugarcane growth and yield worldwide [59].

Recently, Belesini et al. [60] analyzed the transcriptome profiles of the two sugarcane cultivars “SP81-3250” (drought-tolerant) and the “RB855453” (drought-sensitive) under multiple drought stress conditions using Illumina HiScanSQ System and HiSeq 2500 platforms. They found diverse genes that were induced in drought tolerant cultivars, namely ascorbate peroxidase, MYB, E3 SUMO-Protein ligase SIZ2, key enzyme for biosynthesis of flavonoids such as co-enzyme A ligase, and aquaporin [60]. These genes/TFs play important roles in abiotic stress tolerance [61,62,63,64]. Many stress-induced kinases in drought-sensitive cultivars were discovered [60], such as receptor like protein kinases (RLK) which may play a role in the perception of stress stimulus, bHLH transcription factors, 1-aminocyclopropane-1-carboxylate (ACC) oxidase derived from the ethylene biosynthetic pathway, and several undescribed genes. Additionally, Pereira-Santana et al. [65] used a high-throughput sequencing system to analyze the transcriptional profile of the second most important sugarcane variety “Mex 69–290” in Mexico, in response to osmotic stress. They found that the cultivar responded to osmotic stress by increasing gene expression involved in transcriptional regulation, carbohydrate catabolism, oxide-reduction, flavonoid, and other secondary metabolites biosynthesis. Genes responsible for ABA, water deprivation and heat stress were also upregulated.

Another study was reported by da Silva et al. [66] using HT-SuperSAGE technique to evaluate four drought resistant and sensitive cultivars. They identified that 9831 induced unitags from the roots of the tolerant cultivars were different regulation in the sensitive cultivars after stressed by irrigation suppression. Numerous genes play a vital role in the metabolic process of sugarcane, such as ethylene stress attenuation (ACCD), root growth (β-EXP8), protein degradation, oxidative detoxification (TRX), fatty acid synthesis (ACC), amino acid transport (AAT), and carbohydrate metabolism [glycolysis (PFK, TPI, FBA), pentose phosphate pathway (TKT)] [66]. More recently, Liu et al. [67] conducted an experiment on wild type sugarcane “*Saccharum narenga*” treated with drought stress and identified 3389 DEGs (1772 upregulated and 1617 downregulated). These DEGs were involved in such biological pathways as the metabolic pathway, response to blue light, and plant hormone single transduction [67]. Additionally, Vantini et al. [68] conducted a comparative study for screening the gene expression profiles of two different drought-tolerant sugarcane varieties against drought stress through DNA-amplified fragment length polymorphism (AFLP). The AFLP results revealed that 173 fragments of tolerant cultivars have shown the altered expression pattern in response to water stress.

A research on cold stress in sugarcane reported by Selvarajan et al. [69], investigated differential gene expression profiling through transcriptome approach of a cold-tolerant *S. spontaneum* clone “IND 00-1037” under low temperature stress. The DEG analysis shows that 2538 genes were upregulated and 3302 genes were downregulated upon the cold stress. Further investigation revealed that 170 cold responsive transcriptional factors belonged to 30 families, each differently regulated. A DNA binding transcriptional activation protein, CBF6 (C-binding factor), involved in cold acclimation and freezing tolerance. Many cold responsive genes are associated with different metabolic pathways. For example, cold sensing, MAP kinases, phytohormone signaling, calcium, and lipid signaling genes, soluble sugar, lignin and pectin biosynthetic genes were also differentially regulated [69].

Transcriptome profiling of sugarcane in response to low potassium (K) stress and low nitrogen (N) were investigated by Zeng et al. [70] and Yang et al. [71], respectively. Zeng et al. [70] identified a total of 4153 DEGs responding to low-K stress and proposed that transcription factors, transporters, kinases, oxidative stress-related genes and genes in Ca^+^ and ethylene signaling pathways might play crucial roles in improving the tolerance of sugarcane to low-K stress. More recently, Yang et al. [71] reported that MYB was the largest differentially expressed TF gene family in sugarcane varieties ROC22 (low N-tolerant variety) and Badila (low N-sensitive variety) under low nitrogen stress, while some specific DEGs in ROC22 leaves were mainly enriched in photosynthesis and nitrogen metabolism, and some specific DEGs in ROC22 roots mainly enriched in nitrogen metabolism and the hormone pathways.

### 3.3. Transcriptomics Studies on Sugarcane Development/Improvement

The transcriptomic analysis of sugarcane has been carried by applying the efficient and up-to-date molecular techniques, such as cDNA microarrays [72], Roche/454 and Illumina/Solexa [8], RNA-seq, qPCR and microscopy [46], for exploring gene expression profiles from cells/tissues and their effects on structural and functional changes during sugarcane growth and development [73]. The functional characterization and annotation of genes responsible for important agronomic traits are very important and assistive in sugarcane variety improvement for enhancing the productivity. A database (SUCEST-FUN Database) related to the functional genomics of sugarcane was developed to store, retrieve and integrate various types of data, including genome sequencing, transcriptomic, gene expression profiles, gene catalogs, phenotypic records and transgenic plant (http://www.sucest-fun.org/). In addition to ESTs, transcriptomics has been applied to define the expression profiles of genes and to validate the expression patterns in sugarcane [74]. For example, several genes associated with cellulose and lignin biosynthesis [75], leaf abscission [12], ripening [76], and photosynthesis [77] have been identified in agronomic and quality traits and development responses. The transcriptome studies in sugarcane also support the identification of some useful promoters, which can be used in the transgene to control the expression of certain tissue-specific genes. Further to all these efforts, studies are in progress to find out genes ascribed to the traits of interest, including sugar content, tolerance to abiotic and biotic stress for the improvement of sugarcane crop.

## 4. Sugarcane Proteomics

In addition to transcriptome, proteomics approaches also provide new insights into complex biological phenomena [7,78]. Hence, the mechanisms for the quantification of proteins and their post-translational derivatives are fundamental to the study of biological systems. Despite the genome is stagnant, the proteome of an individual dynamically reacts to environmental stimuli and intracellular metabolite levels by variable expression levels and post-translation modifications viz. glycosylation, phosphorylation, methylation, acetylation, etc., further increasing the inherent complexity of proteome [7].

**Figure 3:**
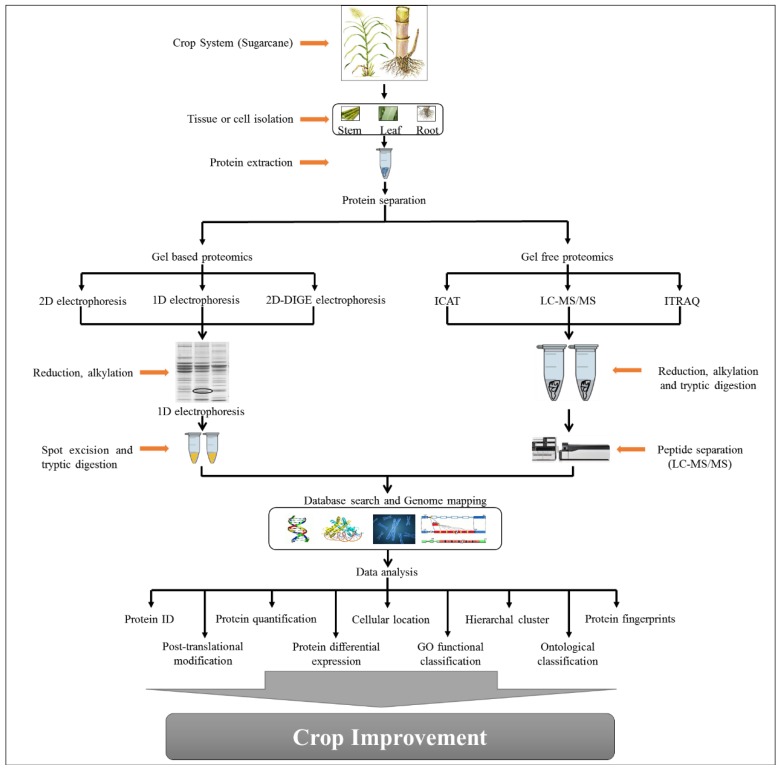
The workflow of sugarcane proteomics from crop system sampling using gel-based and gel-free proteomics approaches.

To determine the differential and comparative expression levels of protein, diverse protein isolation, and quantitation tools, such as two-dimensional electrophoresis (2-DE), mass spectrometry (MS), and matrix-assisted laser desorption/ionization-time of flight mass spectrometry (MALDI-TOF-MS) are used in sugarcane under different biotic and abiotic stresses (reviewed by Barnabas et al. (2015) [7]. Additionally, iTRAQ is one of the major quantitation tools used in differential plant proteomic research [79]. In sugarcane, a number of current proteomic studies have been performed based on gel-based or gel-free tools under different biotic and abiotic stresses. The workflow of sugarcane proteomics from crop system sampling using gel-based and gel-free proteomics approaches is illustrated in Figure 3.

### 4.1. Proteomics Studies on Sugarcane Response to Biotic Stresses

Plant–pathogen interactions are very diverse, while a proteomics-based molecular tool discloses a new perspective of plant defense, together with the pathogenicity and virulence of the pathogens, thereby contributing to a broad understanding of plant diseases [80,81]. In order to elucidate the dynamic molecular events generated during the sugarcane–fungus (*C. falcatum*) interaction process, the stem proteome has been studied in detail, a reference map has been developed [82], and a sensitive staining method with improved MS compliance has been established [83]. Furthermore, the use of two-dimensional electrophoresis-mass spectrometry (2DE-MS) has disrupted the differential regulation of stem and suspension cell proteomes that trigger responses to SAR inducers BTH (benzothiadiazole) and *Cf* (*C. falcatum*) elicitors [84]. A recent secretomics-based study proposed that a cerato-platanin protein (EPL1) of *C. falcatum* identified by 2-DE coupled with MALDI TOF/TOF MS as a potential pathogen-associated molecular pattern (PAMP) was involved in inducing systemic resistance in sugarcane [85].

In order to understand the strategies such as suppress/evade defense mechanisms, proteomics and secretomics-based studies on *S. scitamineum* have been carried out during their interaction with sugarcane resistant and susceptible cultivars. Que et al. [86] applied 2DE and MALDI-TOF-TOF/MS to identify 20 DEPs involved in defense response, signal transduction and photosynthesis during sugarcane response to *S. scitamineum* infection. In addition, Barnabas et al. [87] used the same proteomics technique to investigate the proteome level alterations occurring in the meristem of a *S. scitamineum* infected susceptible sugarcane cultivar at whip emergence stage and suggested that 53 sugarcane proteins identified were related to defense, stress, metabolism, protein folding, energy, and cell division. A more advanced proteomic approach (iTRAQ) was also used in understanding the molecular basis of sugarcane–pathogen interaction [87]. Su et al. [13] identified 273 and 341 differentially expressed proteins in two sugarcane “Yacheng05-179” (smut-resistant) and “ROC22” (smut-susceptible) cultivars. Further analysis shows that most differentially expressed proteins are closely related to sugarcane smut resistance, such as β-1,3-glucanase, endo-1,4-β-xylanase, heat shock proteins, peroxidase, pathogenesis-related protein 1 (PR1) and lectins. The ethylene and gibberellic pathways, phenylpropanoid metabolism and PRs, such as PR1, PR2, PR5 and PR14, are more active in “Yacheng05-179”, suggesting that they may play a role in sugarcane smut resistance [13]. On the other hand, Qi et al. [88] found that the photosynthesis pathway, ROS, ABA, calcium signal pathway related proteins were upregulated in both varieties, GT29 (smut-resistant) and Yacheng 71-374 (smut-susceptible).

Regarding the worldwide ratoon stunting disease of sugarcane, novel insights into the early stages of this disease has been provided by Cia et al. [54] using transcript and protein analysis. Their results show that an increase in the bacterial titers results in variations in the expression of 267 cDNAs and in the abundance of 150 proteins which are involved in hormone metabolism, signal transduction, plant growth, and defense responses. However, some changes are predicted to benefit the pathogen, such as the upregulation of genes involved in the synthesis of methionine. 

### 4.2. Proteomics Studies on Sugarcane Response to Abiotic Stresses

The physiological condition of sugarcane and its growth, metabolism, and development are directly or indirectly affected by several abiotic stresses [7]. Drought is considered “multidimensional stress” because it affects a bunch of cellular processes in crop plants, leading to yield losses [10]. Distinctive variations in the physiological condition of a sugarcane plant under drought stress comprise of high solute concentration as a result of the accumulation of inside cell ions, which afterward outcomes in osmotic imbalance [89]. Plants, under abiotic stresses like cold, drought and salinity, regulate the balance of betaine, sugar, proline and other well-matched solutes together which was termed as osmoprotectants, helping the plant to adapt biochemically to the adverse circumstances [7,89]. The alteration of protein synthesis or degradation is one of the fundamental metabolic processes, affecting drought tolerance.

In order to understand the drought-tolerant mechanisms of sugarcane, proteomics-based studies have been carried out. Sugiharto et al. [90] identified a drought-inducible gene (*SoDip22*) from a drought-stressed cultivar by 2-DE and suggested that *SoDip22* functions to adapt to drought stress in the bundle sheath cell and that the signaling pathway for the induction is, at least in a part, mediated by ABA. A 18KDa protein isolated by the 2-DE method was accumulated in sugarcane leaves under drought stress conditions [91]. Some proteins involved in the photosynthesis pathway and enzymes related to antioxidative damage were separated and identified by 2-DE coupled with LC–ESI–IT–MS/MS [92]. Overexpression of *EaDREB2* (a *DREB* gene isolated from *E. arundinaceus*) and pyramiding of *EaDREB2* with the pea DNA helicase gene (*PDH45*) enhanced drought and salinity tolerance in transgenic sugarcane [11]. To investigate the protein profiles of sugarcane exposed to drought stress, two varieties of sugarcane, RB 72910 (drought-tolerant), and RB 943365 (drought-sensitive) under water deficit for 30 days, and then water stress-related proteins were identified using 2-DE and MALDI-TOF-MS. Some of proteins associated with photosynthesis, signal transduction and regulation process upregulated and downregulated in RB 72910, but those of proteins downregulated in RB 943365. Khueychai et al. [10] isolated and identified various proteins responded to drought in two drought-tolerant (K86-161) and drought-sensitive (B34-164) cultivars by 2-DE coupled with LC–MS/MS. Their results revealed that the expression of fructose-bisphosphate aldolase, oxygen-evolving enhancer protein, and SOD were elevated in two or three organs of K86-161, whereas these proteins decreased in B34-164 under drought stress. More recently, Salvato et al. [93] carried out quantitative proteomics of enriched nuclei from sugarcane (*Saccharum* ssp) stems in response to drought stress by filter-aided sample preparation (FASP) and LC–MS/MS. The results show that most of 74 exclusives proteins of control plants are related to cell wall metabolism, suggesting that drought affects negatively the cell wall metabolism; Also, 37 TFs that were associated to protein domains, such as leucine-rich (bZIP), C2H2, NAC, C3H, LIM, Myb-related, heat shock factor (HSF) and auxin response factor (ARF), are identified. These TFs belong to low abundant nuclear proteins and are differentially accumulated in response to drought stress [93].

Soil salinity is also a limiting factor to sugarcane crop development. To understand the molecular mechanisms of tolerance to salinity stress in sugarcane, Pacheco et al. [94] studied differentially delayed root proteome responses to salt stress in sugarcane varieties using 2-DE and MS analysis (UPLC–ESI–Q-ToF) and revealed that highest accumulation of proteins involved in growth, development, carbohydrate and energy metabolism, reactive oxygen species metabolism, protein protection, and membrane stabilization in a tolerant variety after 2 h of salt stress, whereas the presence of these proteins in a sensitive variety was verified after 72 h of salt stress. Murad et al. [95] carried out an experiment on the proteomic analyses of two frequently used *Saccharum* spp. cultivars (RB867515 and RB855536), grown under salt stress, by 2DE and MS analysis, which reveals that four proteins including fructose 1,6-bisphosphate aldolase, germin-like protein, glyceraldehyde 3-phosphate dehydrogenase, and heat-shock protein 70 were differentially expressed among control and salt-treated genotypes, which confirmed that these genes are involved in the energy metabolism and defense response against salt stress in sugarcane. Passaman et al. [96] analyzed the proteomic effects of salt stress in micropropagated shoots of two sugarcane cultivars (CB38-22 and RB855536) using a label-free proteomic approach (ESI–LC–MS/MS analysis). The results show that a greater abundance of proteins involved in non-enzymatic antioxidant mechanisms, ion transport, and photosynthesis, as well as some proteins (calcium-dependent protein kinase, photosystem I, phospholipase D, and glyceraldehyde-3-phosphate dehydrogenase) were more abundant in the RB855536 cultivar exposure to salt stress. These abovementioned findings indicated that diverse proteins play important roles for the acquisition of ionic and osmotic homeostasis during sugarcane under salt stress.

In addition to drought and salinity stresses, cold is also an abiotic stress affecting sugarcane productivity. Park et al. [97] performed transcriptome analysis of the cold-susceptible cultivar “CP72-1210” and the cold-tolerant “TUS05-05” clone (*S. spontaneum*) under chilling stress, and then showed that the major DEGs between two clones after chilling stress were related to the transmembrane transporter activity. Subsequently, they chose the most important fundamental protein gene (*SspNIP2*) for functional analysis, which showed that transgenic tobacco plants carrying *SspNIP2* gene resulted in more vigorous transgenic lines than the non-transgenic tobacco plants under salt and water stress tolerance. Recently, Chen et al. [98] conducted an experiment on the *NsLTP* gene family, which encodes a protein of 103 amino acid residues, from a full-length cDNA library of sugarcane stem. The *ScNsLTP* transcript levels in sugarcane plantlets reduced in response to salicylic acid (SA), while it enhanced under methyl-jasmonate (MeJA) treatment, signifying an adverse effective mechanism between the signaling molecules of SA and MeJA. Additionally, the transcript levels of *ScNsLTP* were obviously upregulated under low temperature and PEG stresses, suggested that this gene plays a positive role in adaption to chilling and drought stresses [98].

### 4.3. Proteomics Studies on Sugarcane Development/Improvement

Proteomics strategies have also been used to represent proteome regulation through sugarcane development processes along with profiling the proteomes of sugarcane organs. To be well aware of the modeled developmental processes in sugarcane, it is chiefly important to obtain complete proteome features such as high proteome coverage. Until now, several explanatory reports have described characteristics of sugarcane proteomes, including somatic embryogenesis in embryogenic and non-embryogenic callus induced by putrescine [99] and under different red and blue lights [100], cell wall proteomes of different sugarcane organs at two developmental stages [101], and of the sugarcane stem [102], cell wall remodeling in suspension cell [103], and lignin composition in stem development [104]. 

Somatic embryogenesis is an important biotechnological technique, contributing to great potential for application in sugarcane breeding and micropropagation. Thus, Reis et al. [99] evaluated the impact of exogenous polyamines on sugarcane somatic embryo development and modifications in protein excess profiles caused by the effect of 500 μM putrescine on sugarcane somatic embryo development were recorded. Furthermore, proteomic analyses between putrescine and control treatment showed DEPs related to somatic embryogenesis, such as arabinogalactan proteins, peroxidases, heat shock proteins, glutathione S-transferases, late embryogenesis abundant proteins, and 14-3-3 proteins play important roles in protecting the cells against an putrescine-induced stress environment, contributing to the formation of somatic embryos during the maturation treatment. In addition, the combined effect of red and blue lights on sugarcane somatic embryogenesis was revealed by comparative analysis using quantitative shotgun proteomics [100]. Of the 1171 identified proteins, a higher abundance of methyltransferases and clathrin heavy chain 1 in WmBdRfR (450/530/660/735 nm) treatment was related to differentiation and dedifferentiation processes, suggesting that the proteins might be candidate markers for sugarcane somatic embryogenesis.

In plants, cell walls are incessantly modified during plant growth and development, signaling and defense against pathogens, by enzyme actions which are important players in the remodeling of cell wall components. [101,105]. Fonseca et al. [101] characterized the cell wall proteomes of sugarcane young and mature leaves and stems and found that sugarcane leaves and young stems had the highest lipid metabolism (LM) rate than all species and enriched 277 cell wall proteins (CWPs) identified in sugarcane. Calderan-Rodrigues et al. [102] characterized the cell wall proteome of young sugarcane culms, to identify proteins involved in cell wall biogenesis and then identified that 84 different cell wall proteins were related to lipid metabolism and oxido-reductase activity. Another research on the cell wall proteomics of sugarcane cell suspension cultures was conducted by Calderan-Rodrigues et al. [103]. Their study shows that 69 secreted proteins were predicted among 377 proteins and oxidoreductases (such as peroxidases) and were well represented, but glycoside hydrolases were scarce. 

Sugarcane is a promising crop for biofuel production, in addition to producing sugar. Lignin content and composition are the most important factors related to biomass recalcitrance to convert cell wall polysaccharides into fermentable sugars [104]. Salvato et al. [104] reported that lignin arrangement are the reasons of changes in the stalk proteome associated to nitrogen, oxidant metabolism, and carbon, but do not modify lignin content amount. However, from the ~1000 non-redundant proteins known, 28 and 177 were differentially gathered in response to nitrogen and linked with many functional classes, as well as amino acid metabolism, carbon metabolism, oxidative stress, and protein turnover [104].

## 5. Sugarcane Metabolomics 

Metabolomics, the study connecting phenotypic and physiological changes to external stimuli, has become an important element of studying plant biology [106]. It is estimated that the plant kingdom has about >200,000 different metabolites that define the fate of plant yield and quality of produce [107]. This number likely characterizes only a small portion of the global plant metabolic range, and it is estimated that a single plant could be produce up to 15,000 metabolites [108]. Sugarcane metabolomics is still in its early phase because of the complexity in mining molecular markers for important agronomic traits, determining the stable functional molecules and other key components [107]. The determination of different essential metabolites can help in the understanding of sugarcane biology (Table 1). 

**Figure 4:**
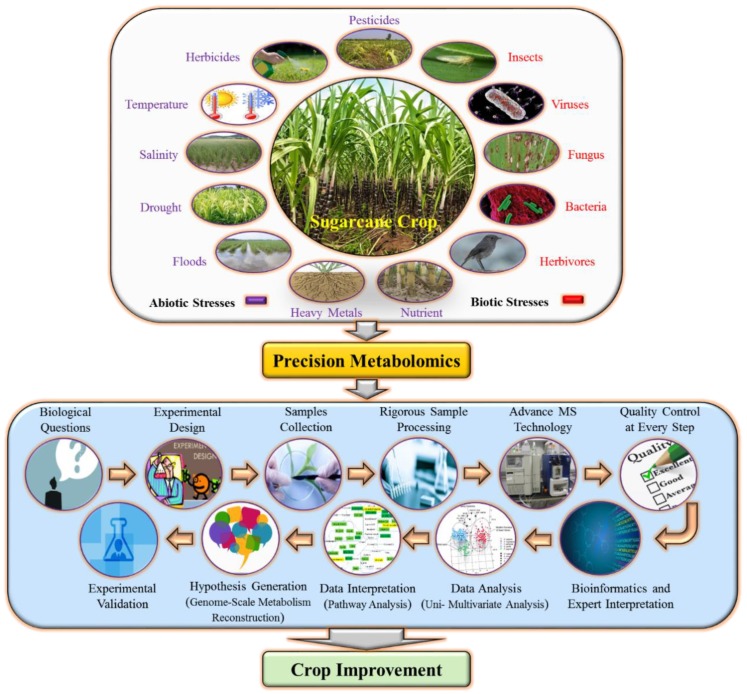
Sugarcane precision metabolomics studies: A useful tool to discover metabolome alterations during abiotic and biotic stresses and major routes of generating metabolomics data.

The determined metabolites were mainly carbohydrates such as sucrose, glucose, fructose, inositol, and raffinose. The researcher used GC–MS analysis with ribitol as an internal standard [109,110]. These carbohydrates metabolites help in the selection of sugarcane genotype with relatively high sucrose content. The application of metabolomics in sugarcane can be of great significance as it can distinguish the similarities between parents and offspring lines [111]. Metabolomics approaches include the employment of mass spectrometry [112], gas chromatography-mass spectrometry (GCMS) [113] and nuclear magnetic resources (NMR) [114] have recently been used by many researchers to determine the primary and secondary metabolites [111,115]. Additionally, these advanced metabolomics tools have been used in sugarcane tissue culture [116]. The study unfolded the connection between callus tissue and media and further revealed that the callus utilizes or secrete the media nutrient [116]. These tools can also be used to examine the effects of environmental adversaries on sugarcane and its produce [117,118]. Further, metabolomics studies are important to unfold the mechanism of sugar accumulation by knowing the interaction between sugar related genes and their resultant proteins in sugarcane [119]. Figure 4 shows a tool to discover metabolome alterations during abiotic and biotic stresses and major routes of generating metabolomics data.

### 5.1. Metabolomics Studies on Sugarcane Response to Biotic Stresses

Metabolomics approaches have been used recently in sugarcane to understand the plant–pathogen interaction [128] and the pathogen hijacked pathways in order to figure out the acquired resistance mechanisms [129]. In line with that, the sugarcane genotypes susceptible to smut disease have been inoculated with *S. scitamineum* SSC39 teliospores [117]. LC–ESI–MS (liquid chromatography coupled to electrospray ionization tandem mass spectrometry) was employed for the purpose to identify the most common molecules which were Apigenin 7-O-(6″-O-acetylglucoside) and 3′-O-methylderhamnosylmaysin across all time points in control and infected plants. The results suggested that during disease propagation, the Apigenin 7-O-(6″-O-acetylglucoside) synthesis was hindered but showed increasing pattern after whip development which makes it an important marker metabolite to identify healthy plants [117]. In another study, the ratoon stunning disease (RSD) triggered by a Gram-positive bacterium known as *Lxx* was used as an inoculant over two sugarcane genotype, resistant and susceptible to this pathogen. The study reveals that, after inoculation, the resistant genotype yielded a high number of phenolic compounds compared to the susceptible one. The general objective of this study is to conclude the metabolic profiles of a “CB49-260” (susceptible) and “SP80-3280” (resistant) varieties inoculated or not with “*Lxx*” and to parallel the results with current proteomics and transcriptomic data to define a basic of targets (genes, proteins and metabolites) that can be verified as markers of resistance in a group of sugarcane varieties [130]. 

Sugarcane yellow leaf virus (SCYLV) causes severe leaf symptoms in sugarcane worldwide. Gonçalves et al. [131] reported that alterations in sugarcane photosynthetic apparatus after plant infection by SCYLV, as showed that a reduction in potential quantum efficiency for photochemistry of photosystem (PSII), alterations in the filling up of the plastoquinone (PQ) pool as well as reduction in the CO_2_ net exchange rates, photosynthetic leaf pigment contents, and ratio of chlorophyll a/chlorophyll b. However, carbohydrate content in the leaves was increased as a secondary effect of the SCYLV infection. In addition, Lehrer et al. [132] reported that SCYLV-infected leaves had a higher level of carbohydrates, especially starch, suggesting a reduction of assimilate export. Similarly, Marquardt et al. [133] illustrated that the photosynthesis and stomatal conductivity were lower on the canopy basis, while the sucrose levels increased in the leaves, reflecting some of the early changes induced in Yellow Canopy Syndrome (YCS) symptomatic plants. They proposed that the first change in metabolism of the YCS symptomatic plants were increased in sucrose contents in leaves, while the other changes are secondary effects caused by sugar levels increment [133]. Subsequently, they investigated the alteration of carbon partitioning in YCS sugarcane. In their study, more than 200 metabolites were detected, but only 84 metabolites were identified. Significant metabolic changes occurred well before the development of leaf yellowing and the main metabolic changes were related with sugar metabolism, pentose phosphate cycle, phenylpropanoid, and α-ketoglutarate metabolism [125]. Altogether, the application of metabolomics approaches could be useful in finding marker metabolites that can help in future sugarcane improvement programs against biotic stress. 

### 5.2. Metabolomics Studies on Sugarcane Response to Abiotic Stresses

In addition to drought and salinity, heat stress is also a main constraint inducing abiotic stress in sugarcane. When sugarcane nodes were soaked in 20 mM proline, the exogenous application of proline protects the sugarcane buds from heat stress by hindering the production of excessive H_2_O_2_ and increased the number of osmolytes along with increased soluble sugar content [124]. Additionally, the exogenous proline also improved the K^+^ and Ca^2+^ and hastened the concentration of free proline content [124]. Rasheed et al. [134] conducted an experiment to explore physiological and developmental variations in the sugarcane immature buds exposed to salt stress. Salinity boost up the generation of hydrogen peroxide, increased tissue levels of Cl^−^ and Na^+^, decreased K^+^ and Ca^2+^, and Ca^2+^: Na^+^ and K^+^: Na^+^ ratios, while increasing the osmolyte synthesis in growing sugarcane buds.

In addition, the sugarcane plants were referred to three water deficit cycles in order to confirm the role of signaling molecules in stress memory mediated resistance in plants [135]. The research stated that the sugarcane plants exposed to the third water deficit cycle exhibited high photosynthetic rate and elongated roots because of the high H_2_O_2_ and MDA activity. The study further suggested that H_2_O_2_ may act as an oxidant and also as a secondary signaling metabolite because of its long half-life and comparatively high penetrability across membranes [136,137]. These biochemical signals proved to be significant in enhancing plant adaptability to drought stress and this method can be applied to raise drought resistance sugarcane plants under nursery conditions [135,136]. 

In other reports, salinity-tolerant and susceptible cultivars were grown under the saline condition to find out the possible metabolites involved in the salinity resistance of tolerant cultivar. A study revealed that salinity tolerant cultivar yielded a high amount of proline content and accumulated lower Na^+^ concentration in the leaf [121]. Similarly, the increased production of proline, soluble phenolic, anthocyanins, and flavones were also attributed to the enhancement of tolerance against drought and salinity stress in sugarcane plants [123,124]. Therefore, it is assumed that the application of metabolomics can be a useful tool to identify stress tolerant genotypes which can provide inroads for sugarcane breeding programs.

### 5.3. Metabolomics Studies on Sugarcane Development/Improvement

The validation and profiling of key metabolic components are helping the plant scientist community to understand the biology of plant development in a more precise manner. In the past several decades, scientists have focused mainly on the processes of sucrose metabolism in sugarcane. GC–MS technology was developed by Bosch et al. (2003) to allow the separation and identification of multiple metabolites [109]. They used this method for a preliminary comparison of metabolite levels in immature and maturing internodes of sugarcane varieties, contributing to understanding of carbon partitioning and sucrose accumulation in sugarcane [109]. Glassop et al. [138] examined the change in metabolite abundance down the stem of sugarcane as sucrose accumulated using GC–MS technology and found several metabolites seemed to be correlated with development and some of metabolites, such as trehalose and raffinose, were positively correlated with sucrose accumulation [138].

In order to bring improvement and development in sugarcane, sink demand for photo-assimilate is the key powerful factor that guides the accumulation of sucrose in growing sugarcane stalk [139]. Due the production of sucrose, transport and accumulation are controlled by many genes and regulatory sequences, so the expression profile and sucrose accumulation patterns of important genes, in both source and sink tissues are desirable to better understand the source–sink relationship [140,141]. To better understand the “source–sink communication”, Roopendra et al. [142] studied the gibberellin-induced perturbation of “source–sink” promotes sucrose accumulation in sugarcane. The study further demonstrated that gibberellin induced enlargement in cell size by 42.3% and in internode length by about 39.3% (sink capacity), decrease sugar level by 177% (sink strength), and improved sucrose-metabolizing enzyme expression (sink demand), which may affect the source–sink dynamics in sugarcane [142,143]. 

Additionally, Verma et al. [144] elaborated that, sucrose synthesis/accumulation is controlled by a number of genes and regulatory sequences as well as three invertases (SAI, CWI, NI), sucrose synthase (SuSy) and sucrose phosphate synthase (SPS). SPS and invertase play key roles in the enhancement of sink strength, ultimately stimulating greater sucrose accumulation in the cane sink tissues. In their study, there was a significant positive correlation found between sucrose (%) of the cane source and sink tissues, as indicated that sucrose (%) increases with maturity, while sugar content declines with crop growth. Similarly, the observed increase in phosphoenolpyruvate carboxylase (PEPC) gene expression supports the fact that higher sink demand results in a greater photosynthetic rate and thus affects the source activity. SPS was found to be active at the initial stages of sugarcane development, signifying its vital role in sucrose synthesis [126,144].

## 6. Current Challenges in Cane Breeding

More recently, sugarcane has been bred mostly for its high sucrose content because sucrose is the main substrate to produce sugar and ethanol. Thus, there has not been a division of sugarcane genotypes since both first-generation technology and raw sugar production, dependent on the amount of sucrose accumulation in the stalk, which is advantageous for the sugarcane industry. However, with the onset of second-generation technology, which now relies on cellulose, sugarcane breeding projects will have to go back to their genetic pools. Traits of high cellulose content in sugarcane tissues will be introduced to bring specific genotypes that use metabolic energy towards the accumulation of either sucrose or cellulose, thus diverging breeding programs in favour of particular goals. Furthermore, including increase in carbohydrate yield, other traits are essential to consider addressing the major challenges in production systems, such as better suitability against biotic and abiotic stresses (pests, diseases, weeds, drought, aluminum toxicity, poor, salinity, cold and compacted soils), flowering, plant potency, and plant architecture, including height, tillering, stalk number, and leaf angle. 

Using omics approaches and modern bioinformatics tools, it has become very easy to annotate the sequences and their regulator mechanism in sugarcane, to better understanding of its genome, genetics, physiology, molecular biology. The regulatory genes for sucrose synthesis and their pathways have been functionally characterized for the allelic variation, copy number, and expression pattern in modern sugarcane cultivars. However, the complex polyploid nature of sugarcane cultivars limits the breeder’s in understanding genotype to phenotype allelic variation and dosage. There is big challenge ahead in elucidation the complete genome sequence of sugarcane due to its complex ploidy and aneuploidy nature. Finally, breeding programs should take advantage of these tools and incorporate them in their selection pipelines to generate superior new cultivars that respond to current and future needs of the industry and the hopes of the general society.

## 7. Conclusions and Perspectives

Sugarcane has all the characteristics of a main raw material for energy, biofuels, and electricity production, and will be used in the bioprocessing and bio-refinery industries. Biotechnology interventions have proven beneficial to more than 18 million growers in 26 countries planting genetically modified (GM) crops with 185.1 million hectares (457.4 million acres) in area, which is increasing every year [7]. Among GM crops, more than 90% of the crops are insect-resistant or herbicide-resistant causes a significant reduction in the use about 37% of chemical pesticide, yields increased by 22%, and growers’ profits rose by 68% [7]. Although biotechnology interventions have produced agronomically enhanced genotypes, scientists are currently making efforts to use sugarcane crops as a platform for the greater production of chemicals with industrial and therapeutic significance. 

Gene discovery by different “omics” methods is vital for sugarcane improvement programs, and useful information about genes unfolded the mechanisms for plant adaptation and responses to biotic and abiotic environments. EST-SSRs have been successfully utilized to understand genetic relationships and genetic diversity. The development of new markers and their incorporation in genetic maps will definitely facilitate programs to accelerate breeding and improvement efforts. Due to the optimization of the transformation technology and the different conditions of the variety, the genetic operation of sugarcane was successful, followed by field trials. 

The current era has observed the advancement of gene silencing or overexpression techniques to study their important function and to generate new and innovative phenotypes that would otherwise be impossible to achieve by traditional methods. The combination of metabolomics research and gene expression research is undoubtedly a potential tool for present and upcoming sugarcane research. Transcriptomic analysis of transgenic plants with different genes of interest will disclose gene regulatory networks linked with necessary agronomic traits. Varieties of sugarcane that are resistant to biotic and abiotic stress are essential for expanding planting in areas; where the environment is challenged. The view of using stress-related genes as markers for breeding or genetic guidance will definitely diminish the environmental impression of sugarcane crops. A better understanding of how sugarcane plants handle stress can help develop cultivars that are suitable for a particular area. Future researches would be significantly improved with a recently discovered genome of the autopolyploid sugarcane *S. spontaneum* [4].

Future directions and expectations should be aimed at cracking the current main hurdles to plants omics. Genotype-phenotypic genetic diversity is linked by improved quantitative and automated selection and screening methods that focus on plant physiology and quality traits. These traits, combined with decision-making algorithms, will enhance the release of newly bred varieties to farmers and avoid long development phases and large-scale field studies. However, for genomics studies related to the genes of interest: since many desired plant traits depend on the interaction of many genes and metabolic pathways with the environment, the enhanced adoption of translational and interactome research with continuously relating molecular data and breeding parameters to field performance, should preferably use more model crop plants. Further, more attention should be given to CRISPR and Epi-genetics molecular events that are evolutionarily most relevant to plant adaptation to changing environments. Finally, the important step towards crop improvement is to promote transparent dialog between molecular biologists and plant physiologists on the one hand and farmers, breeding companies, and the public on the other hand in order to resolve jointly the economic, sociological, legal, and ethical hurdles.

## Figures and Tables

**Table 1:** Summary of sugarcane metabolites and their role in plant growth and stress responses.

Metabolites	Function	Reference
Sucrose, glucose, fructose, inositol and raffinose	Helpful in screening genotypes with high sucrose content	[110]
Apigenin 7-O-(6″-O-acetylglucoside)	Increased susceptibility to *Sporisorium scitamineum* SSC39 teliospores	[117]
Proline	Enhanced resistance against salt stress	[120]
Sodium (Na+)	High production of Na+ content in sugarcane leaves increase susceptibility to salinity stress	[121]
Proline, soluble phenolic, anthocyanins, and flavones	Increased production of these metabolites enhance resistance against drought and salinity stress	[121,122]
K^+^ and Ca^2+^, soluble sugars and proline content	Improved heat stress tolerance	[123]
Sucrose	Helps in sugar metabolism, pentose phosphate cycle, phenylpropanoid and α-ketoglutarate metabolism	[124]
Sucrose, putrescine, glutamate, serine, and myo-inositol	Enhances axillary bud outgrowth	[125]
Ethylene (ET)	Induced sucrose accumulation	[126]
Ascorbic acid (ABA)	Induce proline contents, mitigating salinity stress	[127]
